# Economics of Public Antibiotics Development

**DOI:** 10.3389/fpubh.2020.00161

**Published:** 2020-05-21

**Authors:** Christopher Okhravi

**Affiliations:** Department of Informatics and Media, Uppsala University, Uppsala, Sweden

**Keywords:** antibiotics, policy, interventions, research and development, market entry rewards, direct funding, public pharma

## Abstract

Issuing monetary incentives, such as market entry rewards, to stimulate private firm engagement has been championed as a solution to our urgent need for new antibiotics, but we ask whether it is economically rational to simply take public ownership of antibiotics development instead. We show that the cost of indirectly funding antibiotics development through late phase policy interventions, such as market entry rewards may actually be higher than simple direct funding. This result is reached by running a Monte Carlo simulation comparing the cost of increasing the ratio of investment go-decisions at the outset of pre-clinical development, to the cost of directly funding the same antibiotics under various levels of operational inefficiency. We simulate costs for hypothetical antibiotics targeting six different indications, using data from previous studies. We conclude that while indirect funding may be necessary for the current pipeline we may want to prefer direct funding as a cost effective long-term solution for future antibiotics.

## 1. Introduction

Our healthcare system depend on the availability of antibiotics (1), and while we are witnessing unprecedented progress in the history of antibiotics a lack of funding is still looming (2). A myriad of policy interventions have been suggested (3) and the effects of some even estimated (4, 5). Most monetary interventions are concerned with incentivizing private developers and investors to partake in antibiotics research and development, yet some have begun proposing a transition toward public ownership (6, 7). This study provides initial quantitative justification for these positions by exploring the cost difference between direct funding of antibiotics development and indirect funding in the form of phase and market entry rewards.

We define “direct funding” as the benefactor paying for antibiotics at-cost while either maintaining ownership or not. Direct funding is a common way of funding antibiotics research and development in the early phases of clinical development (8), but isn't commonly applied to commercialization. Direct funding, in our definition, does thus not necessitate public ownership but does require that every project remains fully funded at all times and is never subjected to profitability analysis. Direct funding may thus be the result of some e.g., fully funding grant scheme or public-private partnership. We however assume that some level of inefficiency may, in either case, arise from the planning of such an effort.

We define “indirect funding” as policy interventions that attempt to incentivize private agent participation. Specifically, we investigate prizes. We assume that prizes are non-dilutive and that the beneficiary is obligated to undertake the activities that the prize is intended to support if the prize is awarded before said activities are undertaken. Any remaining funds in case of completion or scientific failure may however be spent at the discretion of the beneficiary. Commonly proposed interventions along these lines are known as phase entry rewards and (partially delinked) market entry rewards.

When issuing early phase prizes for high-risk ventures, such as antibiotics development, the benefactors of said prize may find themselves awarding substantial prizes before ever experiencing a promised product being successfully brought to market. Conversely, when issuing late phase prizes the benefactor might find themselves successfully stimulating many beneficiaries into avoiding financial termination, while rarely experiencing actual expenditures.

A prize won in the early phases of a venture will, due to the time value of money, improve the venture's financial attractiveness significantly more than a prize of the same size won many years down the line. When paying early, the size of a prize can be smaller as a beneficiary with a high time preference will value the prize higher. However, as the benefactor bears the cumulative cost of many failed ventures it must also afford spreading its capital across many investments to increase its chances of seeing successful ventures reach the market. When paying later, the size of a prize must be higher since a beneficiary with a high time preference values the prize lower. However, as the beneficiary now bears the cost of possible failure, the prize must be significantly greater in order to remain attractive. Due to the high risk of failure, the benefactor may however afford to promise higher prizes.

This juxtaposition provokes the question of when a prize should be awarded to an antibiotic developer in order to strike a balance between efficient use of public funds and successful stimulation of private investments. Or even under what conditions it is economically preferable for the benefactor to directly fund antibiotics development by paying at-cost rather than indirectly funding by issuing prizes.

## 2. Overview

The paper is structured as follows. First we show how the financial valuation method known as expected net present value (ENPV) not only can be used to evaluate private firm go-/no-decisions but also the expected, capitalized costs of both indirect and direct funding. We then present how we've sourced the simulation input data from Sertkaya et al. (4), and then extend it with our assumptions of a social discount rate based on Moore et al. (9) and an operating inefficiency, both representative of a public organization. We then show how the conditional probability of turning a pre-clinical no-decision into a go-decision, P(go), is correlated to prize size, using logistic regression. We use this model to solve for prize sizes that yield a range of P(go) and then combine these prize values with a range of operating inefficiencies and rerun the simulation for each combination. From this we build a heatmap of the mean cost difference in all simulated combinations of P(go) and operating inefficiency. We then highlight the break points where direct funding become cheaper than indirect funding. Lastly, we run the simulation a third time to explore the costs and cost savings of direct and indirect funding in a specific scenario where operating inefficiency is 50% and prizes set to sizes that yield a P(go) of 90%.

## 3. Valuation Method

We Monte Carlo simulate go-/no-decisions, at the outset of pre-clinical development, of a large number of hypothetical antibiotics projects, targeting six indications, under three conditions: no intervention, indirect funding, and direct funding. Practically this results in more than two treatment groups as we explore prizes in phases 1–4, and market entry. We thus have six data sets (the indications) and six treatments (direct funding and indirect funding at five different phases). The term phase 4 is here used to refer to all activities between the end of phase 3 and the first year of sales.

To compare the cost of direct and indirect funding we must quantify. For indirect funding, we need a way to determine which projects will reach a go-decision before and after a given prize intervention as this will determine which projects the benefactor actually might pay for. Further, many argue that planning without respecting market logic inherently entail inefficiencies and for this reason we need a way to compute the cost of indirect funding under different inefficiencies. This leads us to four valuation perspectives: (1) private value, (2) intervened private value, (3) indirect cost, and (4) direct cost. Private value is the value the private owner assigns the project and in turn use as a basis for a go-/no-decision. Intervened private value is that same valuation method applied while considering some publicly announced prize. Indirect cost is the benefactor's cost of announcing said prize, while direct cost is the benefactor's cost of simply paying for the project at-cost.

Pharmaceutical developers have been known to (10) base go-/no-decisions on expected net present value. This financial metric is suitable not only for estimating go-/no-decisions but also for computing intervention costs from the benefactor's perspective since it considers both the opportunity cost of capital and the risk of project failure at various points in time. Therefore we compute ENPV from all four perspectives above, resulting in: (1) private ENPV, (2) intervened private ENPV, (3) indirect ENPV, and (4) direct ENPV. We compute private ENPV as:

(1) $$\begin{array}{l} \underset{t\in T}{\sum }\frac{\left(R_{t}-C_{t}\right)P_{0}}{{\left(1+r\right)}^{t}P_{t}} \end{array}$$

where *T* is the set of all time steps of the project (i.e., all development and market years), *r* is the discount rate of the evaluating private agent, *R*_*t*_−*C*_*t*_ is the cashflow at time step *t* computed by subtracting costs from revenues. *P*_0_ is the probability of reaching the market from the point (in our case pre-clinical) at which ENPV is calculated, *P*_*t*_ is the probability of reaching the market from the entry point of time step *t* which means that *P*_0_/*P*_*t*_ is equivalent to the probability of completing time step *t*−1. We assume that an agent will reach a go-decision without an intervention if private ENPV ≥0, and with an intervention if intervened private ENPV ≥0. We compute private intervened ENPV as:

(2) $$\begin{array}{l} \underset{t\in T}{\sum }\frac{\left(R_{t}-C_{t}+Z_{t}\right)P_{0}}{{\left(1+r\right)}^{t}P_{t}} \end{array}$$

where *Z*_*t*_ is the prize (if any) associated with time step *t*. We compute indirect ENPV as:

(3) $$\begin{array}{l} \underset{t\in T}{\sum }\frac{-Z_{t}P_{0}}{{\left(1+r^{\prime }\right)}^{t}P_{t}} \end{array}$$

where the only considered cashflow is the issuing of the prize, and *r*′ is the discount rate of the benefactor, i.e., of a potentially public agent. Lastly, we compute direct ENPV as:

(4) $$\begin{array}{l} \underset{t\in T}{\sum }\frac{-\left(1+i\right)C_{t}P_{0}}{{\left(1+r^{\prime }\right)}^{t}P_{t}} \end{array}$$

where *i* is the inefficiency fraction of the direct funding agency. In this last case only costs and no revenues or prizes are considered. Note that only the contents of the cashflows and the level of discounting is changed between perspectives. Also note that indirect and direct ENPV are both always ≤ 0 as they only consider costs.

## 4. Input Data

The simulation input data is adapted from Sertkaya et al. (4) and describe hypothetical antibiotics targeting the following six indications: acute bacterial otitis media (ABOM), acute bacterial skin and skin structure infections (ABSSSI), community acquired bacterial pneumonia (CABP), complicated intra-abdominal infections (CIAI), complicated urinary tract infections (CUTI), and hospital acquired/ventilator associated bacterial pneumonia (HABP/VABP). Development cost, time, and probability of success is applied by phase and reported in Table 1. The term “mid” is consistently used to refer to the most likely value of a triangular distribution.

**Table 1:** Development time (months), cost (million USD), and probability of success (%).

**Phase**	**Indication**	**Time min**	**Time mid**	**Time max**	**Cost min**	**Cost mid**	**Cost max**	**Prob min**	**Prob mid**	**Prob max**
PC	ABOM	52	66	72	19	21.1	23.2	17.5	35.2	69
	ABSSSI	52	66	72	19	21.1	23.2	17.5	35.2	69
	CABP	52	66	72	19	21.1	23.2	17.5	35.2	69
	CIAI	52	66	72	19	21.1	23.2	17.5	35.2	69
	CUTI	52	66	72	19	21.1	23.2	17.5	35.2	69
	HABP/VABP	52	66	72	19	21.1	23.2	17.5	35.2	69
P1	ABOM	9	10.5	21.6	7.3	9.7	12	25	33	83.7
	ABSSSI	9	10.5	21.6	7.3	9.7	12	25	33	83.7
	CABP	9	10.5	21.6	7.3	9.7	12	25	33	83.7
	CIAI	9	10.5	21.6	7.3	9.7	12	25	33	83.7
	CUTI	9	10.5	21.6	7.3	9.7	12	25	33	83.7
	HABP/VABP	9	10.5	21.6	7.3	9.7	12	25	33	83.7
P2	ABOM	12	15	30	7.4	9.2	11	34	50	74
	ABSSSI	9	10	30	7.12	8.9	10.68	34	50	74
	CABP	12	15	30	7.28	9.1	10.92	34	50	74
	CIAI	10	11	30	7.68	9.6	11.52	34	50	74
	CUTI	10	11	30	7.28	9.1	10.92	34	50	74
	HABP/VABP	16	18	30	12.48	15.6	18.72	34	50	74
P3	ABOM	20	24	47	33.36	41.7	50.04	31.4	67	78.6
	ABSSSI	10	12.5	47	26.88	33.6	40.32	31.4	67	78.6
	CABP	10	12.5	47	31.04	38.8	46.56	31.4	67	78.6
	CIAI	17	21.5	47	40.48	50.6	60.72	31.4	67	78.6
	CUTI	17	21.5	47	35.04	43.8	52.56	31.4	67	78.6
	HABP/VABP	35	39	47	81.12	101.4	121.68	31.4	67	78.6
P4	ABOM	6	9	12.5	–	1.9588	–	83	85	99
	ABSSSI	6	9	12.5	–	1.9588	–	83	85	99
	CABP	6	9	12.5	–	1.9588	–	83	85	99
	CIAI	6	9	12.5	–	1.9588	–	83	85	99
	CUTI	6	9	12.5	–	1.9588	–	83	85	99
	HABP/VABP	6	9	12.5	–	1.9588	–	83	85	99

Sales data is a combination of total market size (Table 2), market share (Table 3) and product launch success probability (Table 4). In line with Sertkaya et al. (4) we do not directly sample the market share distribution, but rather the product launch success probability, and then apply that sample to reach an estimate for every year's market share. This ensures that the market share does not vary widely between the lower and upper bound on a yearly basis. Instead, the point between the lower and upper bound remains constant, while the lower and upper bounds instead themselves vary. This also ensures that no year has a lower market share than the previous year before peak year sales.

**Table 2:** Market size (million USD).

**Indication**	**Min**	**Max**
ABOM	2,720	
ABSSSI	3,070	
CABP	2,290	9,230
CIAI	2,530	
CUTI	5,760	
HABP/VABP	1,780	

**Table 3:** Market share (%).

**Years**	**Min**	**Max**
1	0.05	0.11
2	0.87	1.91
3	1.57	3.47
4	2.57	5.68
5	3.92	8.64
6	5.79	12.77
7	7.52	16.59
8	8.52	18.80
9	10.10	22.30
10:20	12.27	27.08

**Table 4:** Additional parameters.

**Parameter**	**Min**	**Mid**	**Max**
Launch success probability (%)	40	60	80
Generic entry revenue reduction (%)	25	50	75
Generic entry (years)	10	12	14
Private discount rate (%)	9	11	24

Additional parameters are reported in Table 4. In line with Sertkaya et al. (4) we assume a total product life (i.e., market life) of 20 years. Also in line, we assume that patent expiry leads to a reduction in revenues due to generic entry. This means that the captured market share will increase from year 1 to 10, and then remain constant until generic entry (i.e., patent expiry), upon which it will be reduced to a lower constant until year 20. Table 4 also reports the opportunity discount rate or opportunity cost of capital employed by Sertkaya et al. (4).

Beyond phase-specific development costs, Sertkaya et al. (4) also considers costs for additional supply chain activities, non-clinical work, and post-approval studies (PAS). The costs are spread across various phases as indicated by Table 5. Sertkaya et al. (4) report that the cost of post-approval studies may last up to 3 years following market entry and we assume that it is evenly distributed over exactly 3 years.

**Table 5:** Additional costs (million USD).

**Activity**	**Min**	**Mid**	**Max**	**Spread across**
Sample prep.	2.4	2.7	2.9	P1, P2, P3
Process dev.	18.7	26.8	34.8	P1, P2
Plant design	10.7	13.4	16.1	P3 (75%), P4 (25%)
Plant build	69.6	83	96.3	P4
Non-clinical	3.4	3.7	4	P2, P3, P4
PAS	8	10	12	M1, M2, M3

### 4.1. Our Assumptions

Moving from the data sourced from Sertkaya et al. (4) to our own assumptions, the interventions we model can be viewed as voluntary and non-dilutive prizes. These interventions are akin to what is commonly referred to as a market entry reward (if the prize phase is market entry) or a phase entry reward (if the prize phase is a development phase). We model the prize phase of an intervention as a categorical variable and prize size as a numeric variable.

Prize size is sampled logarithmically on the form 10^*X*^ where *X* is a random variable uniformly distributed between 5 and 11. We sample logarithmically to reduce the required sample size as we've observed that the majority of the effect on P(go) occurs at low prize sizes while substantially higher prizes are required for P(go) to ~1. The sampled prizes may thus range from 100,000 to 100 billion USD. We explore prizes awarded upon entry into phases 1–4, as well as the market but never combine multiple prizes. We do not consider pre-clinical entry prizes as they would suffer from no discounting in ENPV given that the viewpoint from which we evaluate projects is pre-clinical.

We assume that plan based as opposed to free market based operations entail some inherent inefficiency. We explore inefficiencies from 0 to 100% and assume that inefficiency affects cost but neither time of development nor probability of success. We thus assume that it costs more for a direct funding agency to achieve an equivalent probability of success within the same time frame. Inefficient cost is, as can be seen in Equation (4), computed as (1+*i*)*C*_*t*_ where *i* is the fraction of inefficiency and *C*_*t*_ is the cost of time step *t*.

Direct and indirect ENPV, given in Equations (3) and (4), both consider a benefactor specific discount rate *r*′ that is not necessarily equal to the private discount rate *r* reported in Table 4. We assume that the benefactor is a public sector like actor, and as such we uniformly distribute the, so called, social discount rate between 3.5% and 4.5%. The former is the recommendation of Moore et al. (9) and the latter our effort to err on the right side. Moore et al. (9) offer other numbers for intragenerational projects and projects that “crowd out” private investment. However, while the benefits of a new antibiotic may span multiple generations, all costs should be incurred within a single one. Since we're computing the within-subject cost difference between indirect and direct funding, the benefits can be set to zero, meaning that we assume that the benefits derived from directly funding an antibiotic are equivalent to those of indirectly funding that same antibiotic. Consequently, we use a social discount rate appropriate for generational projects. Lastly, since the very reason for the incentive debate is a lack of private investments, “crowding out” ought to be considered a non-issue.

To compute ENPV we must make an assumption about how a real world evaluator of a project periodizes future costs, revenues and probabilities. In other words, how to transform a sequence of discrete phases into a sequence of discrete and uncertain cashflows. We assume that the evaluator converts every phase into a series of years and evenly (i.e., constantly) distribute all cashflows and probabilities of success over the years within a phase. Note that even if some additional costs reported in Table 5 are evenly distributed across some phases they might not be evenly distributed over years as not all phases are equally long. Also note that the generated data points are equidistant within a phase but not necessarily across. If e.g., the duration of PC is 2.5 years and P1 is 1.75 years then we generate 3 data points corresponding to *t* = 0, *t* = 1, and *t* = 2, for PC and then 2 data points for P1 corresponding to *t* = 2.5 and *t* = 3.5. P2 will then start at *t* = 4.25 (because 2.5+1.75 = 4.25). We assume that prizes are executed as one-time lump-sum payments upon phase entry.

### 4.2. Limitations

We do not consider the public cost of drug reimbursement which in reality would inflate the cost of indirect funding. We also do not consider the reduction of development costs associated with already existing grants for antibiotics development. Such grants would reduce private cost and hence improve private ENPV, which in turn would deflate the cost of indirect funding. If such grants however are paid by the benefactor then they would also deflate the cost of direct funding, as the grants no longer have to be paid.

Drug reimbursement and grants are both health care system specific implementation details. While there are extensive studies, e.g., Savic and Årdal (11) on grants, taking these numbers into consideration here would overspecialize the results, multiply the number of input dimensions and assumptions, and thus increase uncertainty. This study elucidates the relationship between non-system-specific parameters. We do however encourage future studies to explore the implications of grants and reimbursements on cost-savings.

Lastly, we do not, in direct funding, consider the possible cost of acquiring a new drug candidate entering into pre-clinical. This is relevant if the candidate is already considered intellectual property and the owner is seeking monetary compensation. A new candidate with a favorable market value is not in need of an intervention and will proceed irrespectively. A new candidate with an unfavorable market value does however need an intervention, but demanding a prohibitively large price for it is questionable given the seller's lack of alternatives. Nevertheless, it has been argued (12) that the societal value of antibiotics is starkly different from its market value. If a new candidate is desperately needed, and the direct funder is the public sector, then the candidate owner might leverage our dire need to drive up the acquisition price. While in such situations, cost-efficiency ought to be of lesser importance, we deem this a valuable avenue for future research.

## 5. Predicting go-Decisions

Running the simulation at 2,000 sampled projects per indication we reach the results reported in this section. Assuming that go-decisions are only reached by private developers when private ENPV ≥ 0, and dividing the number of go-decisions by the total number of projects sampled we get the baseline probability of go as reported in Table 6.

**Table 6:** Probability (%) of go/no-decisions before interventions.

**Indication**	**Go**	**No**
CUTI	89	11
ABSSSI	83	17
CIAI	77	23
ABOM	75	25
CABP	75	25
HABP/VABP	60	40

As the modeled prize interventions, for our intents and purposes, have no strings attached, they always improve ENPV. We can compute this improvement in ENPV by subtracting private ENPV from intervened private ENPV. ENPV improvement is correlated to prize size, but due to heteroscedasticity in improvement with respect to prize size we choose to predict the *log*_10_ transformed difference from the *log*_10_ transformed prize size. We apply the model:

(5) $$\begin{array}{l} log_{10}\left(improvement\right)=\beta_{0}+\beta_{1}log_{10}\left(prize\text{\_}size\right) \end{array}$$

to each indication and prize phase combination separately to avoid having to include further dependent variables. The highest resulting *p*-value is lower than 10^−319^.

**Figure 1:**
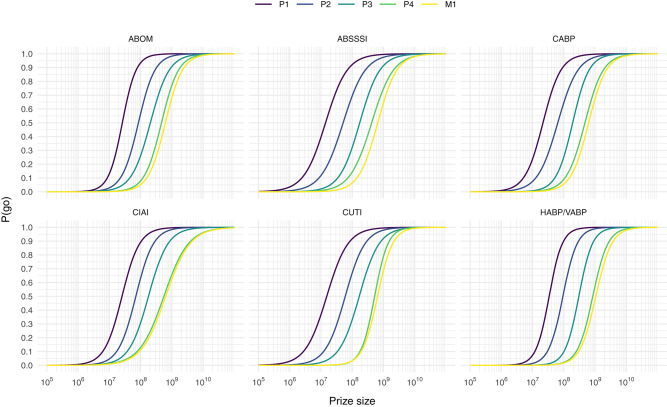
Predicting the conditional probability of turning a no-decision into a go-decision, P(go), from prize size for each prize phase and indication combination.

Since private ENPV improvement is correlated to prize size, and since binary go-/no-decisions are consequences of private ENPV, go/no must be correlated to prize size. To predict the probability of go, call it P(go), we apply the following logistic regression model:

(6) $$\begin{array}{l} ln\frac{P\left(go\right)}{1-P\left(go\right)}=\beta_{0}+\beta_{1}\;log_{10}\left(prize\text{\_}size\right) \end{array}$$

to each indication and prize phase separately, and get a highest *p*-value lower than 10^−6^. Note that we only apply the model to data points where a go-decision was not reached before the introduction of the intervention (i.e., where private ENPV <0). This model does thus not predict the general probability of go under some prize size, but the conditional probability of turning a no-decision into a go-decision, given a project facing a no-decision. Predicting P(go) from our sampled prize sizes yield the sigmoid curves depicted in Figure 1. From this model of the log-odds we can of course predict P(go):

(7) $$\begin{array}{l} P\left(go\right)=\frac{e^{\beta_{0}+\beta_{1}\;log_{10}\left(prize\text{\_}size\right)}}{e^{\beta_{0}+\beta_{1}\;log_{10}\left(prize\text{\_}size\right)}+1} \end{array}$$

and also solve for prize size:

(8) $$\begin{array}{l} log_{10}\left(prize\text{\_}size\right)=\frac{ln\left(\frac{P\left(go\right)}{1-P\left(go\right)}\right)-\beta_{0}}{\beta_{1}} \end{array}$$

which allows us to predict the prize size required to achieve some target P(go).

If the intent of a policy maker is to turn the vast majority of no-decisions into go-decisions without wasting too much money on firms with extraordinary profit requirements we assume that a P(go) of 90% is a reasonable target given the curves in Figure 1. Using Equation (8) we can compute prizes that would yield such a P(go) for every indication and prize phase combination (see Table 7).

**Table 7:** Prizes yielding P(go) = 90% (million USD).

**Indication**	**P1**	**P2**	**P3**	**P4**	**M1**
ABOM	79	323	940	1,841	2,456
ABSSSI	81	305	884	2,432	3,066
CABP	92	326	719	2,083	2,598
CIAI	105	291	863	4,008	4,494
CUTI	85	295	840	1,533	2,188
HABP/VABP	98	295	907	2,859	3,800
Min	79	291	719	1,533	2,188
Max	105	326	940	4,008	4,494

Consistent with previous works (4), we observe that late phase rewards must be substantially larger to achieve the same improvement in ENPV and thereby in probability of go. Previous works have also highlighted the issues of over-incentivizing (13) or overspending (14), defined as paying more than necessary, by (possibly inadvertantly) aiming for certainty of go rather than cost-efficiency per go. Table 7 makes it clear that significant overspending may also occur if target indication is not considered in prize size design, as the difference between the highest and lowest prize of a market entry reward yielding a P(go) of 90% is around 2.3 billion USD.

## 6. Estimating Cost Savings

When estimating the cost of indirect and direct funding in order to compute the within-subject difference between the two, we choose to remove samples with intervened private ENPV <0. Computing indirect ENPV for a project where the intervention is unable to stimulate a private developer to reach a go-decision is misleading as the cost for the benefactor never would be incurred. The cost of an ineffective indirect intervention is thus either zero or meaningless. As both indirect and direct ENPV only consider costs they will necessarily be negative (or zero). When subtracting indirect ENPV from direct ENPV, a positive number thus reflects that the direct alternative is cheaper, and vice versa. We will refer to this number as the cost savings of direct funding.

The cost savings per sampled project can be thought of as the cost savings per go-decision. Not meaning the cost of generating a go-decision, but rather the cost that the benefactor is expected to expend for every observed go-decision. By dividing the indirect and direct ENPV of a given project by the (technical) probability that it reaches market, we transform the expected cost (and hence cost savings) per go-decision into an expected cost (and cost savings) per market approval. Meaning that it answers the question of how much the benefactor is expected to expend for every observed market approval. We prefer this figure due to its simpler interpretation.

Cost difference depends on both benefactor inefficiency and prize size, since inefficiency affects direct ENPV (by increasing development cost) and prize size affects indirect ENPV (as it is the only cashflow considered). As P(go) is correlated to prize size, we could say that cost difference depends on P(go) rather than prize size. However, while (not shown in this paper) the effect of inefficiency on direct ENPV is linear, the effect of P(go), our proxy for prize size, on indirect ENPV is non-linear (also not shown). Consequently, the combined effect of P(go) and inefficiency on cost difference must be non-linear. While the modeling of actual cost difference is the subject of ongoing work, we here rerun the simulation at 10 samples while holding every combination of 40 P(go) values between 0.5 and 0.9875, and 41 inefficiencies between 0 and 1 constant.

**Figure 2:**
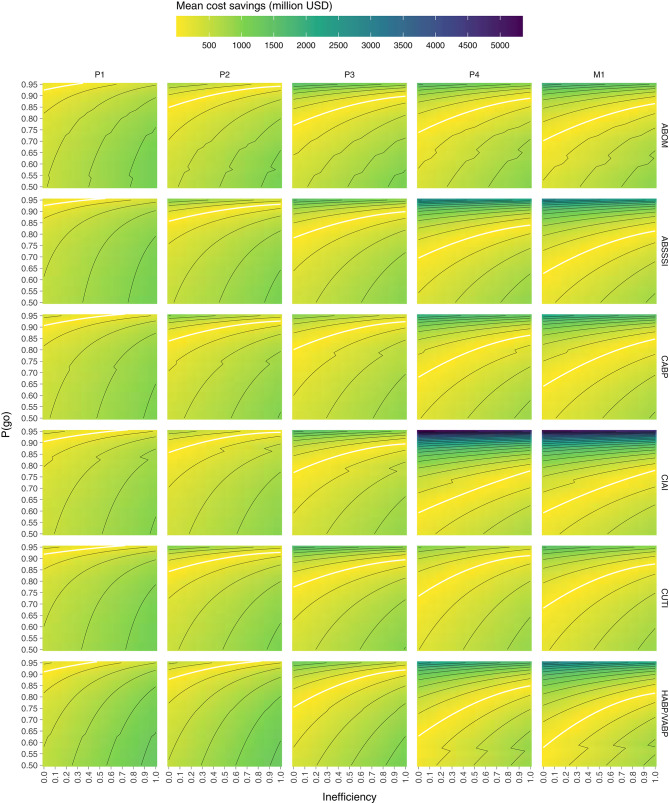
Mean cost savings of direct funding (above the white line) and indirect funding (below the white line) per combination of P(go) and direct funding inefficiency. Lines delimit bins of 250 million.

**Figure 3:**
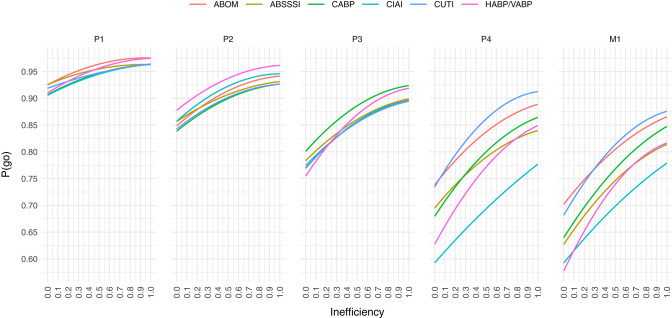
At a given public inefficiency (x), the corresponding P(go) (y), is the maximum P(go) that a prize can target before direct funding is equally expensive. Below each line, indirect funding is on average cheaper per market entry, while above the line direct funding is.

Computing the mean cost savings per combination of P(go) and inefficiency we get the results visualized in Figure 2, where values above the white line denote cost savings of direct funding while values below denote cost savings of indirect funding. The white line is a quadratic polynomial fitted against the points closest to a cost difference of zero. By only plotting these fitted lines in Figure 3 we are more easily able to estimate the maximum P(go) a prize size can target, under some assumed inefficiency before direct funding becomes equivalently expensive and eventually cheaper. Note that the maximum P(go) plotted in Figure 2 is 95%, since higher P(go) values cause a drastic increase in cost difference which renders the heatmap useless.

To summarize Figure 2, indirect funding through phase 1 rewards is always cheaper than direct funding unless the target P(go) is very high (>90%) and inefficiency is low, but even then the cost savings are comparatively moderate. Indirect funding through phase 2 rewards can however be more expensive than direct funding even at an inefficiency of 100% for all indications except one (HABP/VABP). While the cost savings may appear moderate, they do in the worst-case [i.e., high P(go) and low inefficiency] of some indications (ABSSSI, CABP, CUTI) exceed 750 billion USD per market entry. Indirect funding through phase 3 rewards starts becoming quite expensive if inefficiency is low or P(go) is high. The cost savings in the worst-case exceed 1 billion USD in all indications but one (CABP). At phase 4 and market entry, indirect funding is significantly more expensive unless inefficiency is very high or target P(go) very low. At a P(go) of 95% the cost savings are in some indications as high as 2–5 billion USD per market entry.

Figure 3 shows that we are unable to achieve a P(go) of 95% with phase 4 prizes and 90% with market prizes, without the mean costs exceeding those of direct funding, in all inefficiencies and for all target indications. In the indication with the highest cost savings (CIAI) we aren't even able to generate a P(go) of 80% without exceeding the costs of direct funding.

Generally, the color scale (z-axis) of Figure 2 makes it evident that, given the input parameters considered, the possible positive cost savings are significantly greater than the possible negative ones (~5 × ). Granted, we are not looking at the cost savings at P(go) <50% but at such low targets we may wish to question the purpose of a policy intervention.

## 7. Scenario

While Figures 2 and 3 give an indication of how cost savings are correlated to P(go) and inefficiency, to concretize these general results we rerun the simulation at 1,000 samples while holding prize size constant at rates that yield P(go) = 90% (for each prize phase) and inefficiency at 50%. This, we argue, is a reasonable target if the purpose of an intervention is to avoid termination of antibiotics projects for financial reasons, and a reasonable level of inefficiency following direct funding.

**Figure 4:**
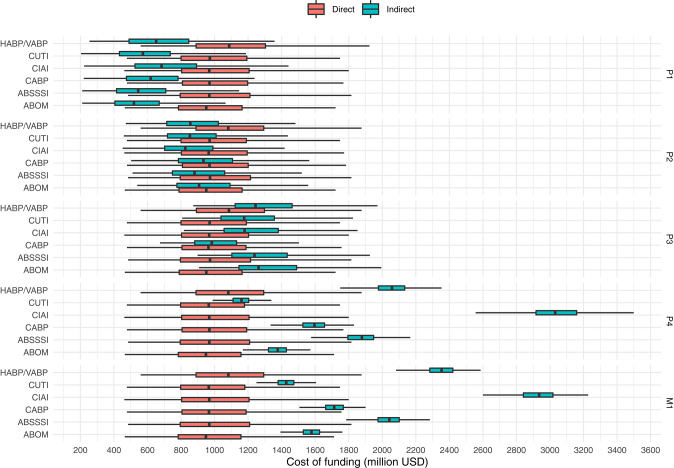
Costs per market entry (in million USD) of indirect funding (at prize sizes that yield a P(go) of 90%) and direct funding of the same projects (at a benefactor inefficiency of 50%). Outliers not shown.

Figure 3 suggests that indirect funding in this scenario should be cheaper when prizes are awarded in phase 1 and 2, but more expensive when awarded in later phases. Indeed this is supported by the final run of the simulation, as can be observed in both, Figure 4 which plots the distribution of funding costs per market entry and in Table 8 which reports the mean cost savings of direct funding per market entry.

**Table 8:** Mean cost savings of direct funding (in million USD), per market entry, at a benefactor inefficiency of 50%, over indirect funding with prize sizes that yield P(go) = 90%.

Indication	**P1**	**P2**	**P3**	**P4**	**M1**
ABOM	−448	−47	336	372	576
ABSSSI	−451	−118	258	837	1,001
CABP	−371	−57	15	570	692
CIAI	−297	−163	215	1,993	1,894
CUTI	−421	−141	200	137	402
HABP/VABP	−438	−238	188	928	1,225
Min	−451	−238	15	137	402
Max	−297	−47	336	1,993	1,894

Figure 4 shows that the cost of direct funding remains fairly constant even though the underlying distribution of projects to fund may slightly vary from phase to phase as a consequence of the subset of projects that are enticed to a go-decision by the indirect funding scheme in question. We observe how the costs of indirect funding steadily rise as prizes are paid in later phases. However, phase 4 prizes, as opposed to market entry prizes, seems to be the most expensive solution for one indication (namely CIAI). Interestingly, the cost differences between indications are inflated with late phase funding (as compared to early phase funding) which again highlights the non-triviality of designing a “one size fits all” prize that doesn't cause overspending. Lastly, while we again observe that the possible range of cost savings in late phase direct funding greatly outweigh that of indirect early phase funding, early phase prizes, still remain the cheapest option in the simulated scenario.

## 8. Discussion

While this study suggests that indirect funding through early phase prizes seems considerably cheaper than direct funding, there's great risks involved for the benefactor in “buying the pig in the poke.” Previous works have pointed to principal-agent problems when issuing large early phase rewards, as there is a risk that agents would abandon development after receiving funding (4).

However, while early phase prizes are too uncertain, we show that late phase prizes are too expensive. Whether issuing market entry rewards ranging up to ~4.5 billion USD is feasible or not is ultimately a policy question. Yet, paying an additional ~1.9 billion USD per approved antibiotic, by issuing a broad market entry or phase 4 reward instead of directly funding the same antibiotics, we find hard to justify.

Rome and Kesselheim (15) raised concerns around the societal costs of market exclusivity vouchers for antibiotics, but was met with critique from Boucher et al. (16) due to neither suggesting alternatives nor taking the societal value into account. We appreciate the inviability of maintaining “business as usual” (17), and the urgent need for sustained innovation, but suggest that a cheaper alternative to market oriented interventions might simply be to pay at-cost.

Financial rewards following market exclusivity vouchers have, according to Rome and Kesselheim (15), varied widely without being related to a drug's societal value or innovativeness. Indirect funding in this study takes neither societal value nor innovativeness into consideration, so while early phase prizes might be a cost-effective solution when compared to direct funding, “rewarding all drugs with the same payments could create perverse incentives to produce drugs that provide the least possible innovation” (18). Direct funding alleviates this problem altogether by, apart from candidate acquisition, side-stepping market logic.

Finding a causal model for evidence-based policy, in the vein of Cartwright and Stegenga (19), may require modeling antibiotics research, development, consumption, and bacteria transmission as a complex adaptive system. Predictions would be rife with error. See e.g., Almagor et al. (20) for a sophisticated model of the impact of antibiotics use on transmission of resistant bacteria in hospitals alone. It may thus be wise to delink our need for antibiotics from the uncertainty of the free market and thereby reduce rather than increase complexity. Especially, if direct funding in fact may be cheaper.

While the results of this study are tightly coupled to the input parameters considered, we urge the community to suggest additional or alternative parameters that, if they exist, support an opposing view of the one presented. If we can save up to almost two billion USD per market approved antibiotic while also retaining IP and thus reap further benefits, such as full control of drug pricing and distribution meaning the ability to deploy access and stewardship measures without having to cater to the profit requirements of private firms, there is ample reason for further investigation.

These results clearly indicate that there is rational, economic evidence supporting the idea of direct funding of development (possibly in the form of public pharma) in the case of antibiotics. As emphasized by Singer et al. (6), this is not a radical suggestion. Building on their proposal of a two-pronged approach, indirect funding could be used as a short-term solution to extract the antibiotics currently in the pipeline, while direct funding would be used to secure cost-effective long-term access to new antibiotics.

## Data Availability Statement

The datasets generated for this study are available on request to the corresponding author.

## Author Contributions

The author confirms being the sole contributor of this work and has approved it for publication.

## Conflict of Interest

The author declares that the research was conducted in the absence of any commercial or financial relationships that could be construed as a potential conflict of interest.
